# Supplementary material for: Long-term cargo tracking reveals intricate trafficking through active cytoskeletal networks in the crowded cellular environment
Source: Nat Commun. 2023 Nov 14;14:7160. doi: 10.1038/s41467-023-42347-7 (PMC10645962; doi:10.1038/s41467-023-42347-7)
Supplement: Supplementary file 1 — Supplementary Information [file 41467_2023_42347_MOESM1_ESM.pdf]

Supplementary Information for

Long-term cargo tracking reveals intricate trafficking through active cytoskeletal networks in the crowded cellular environment

Jin-Sung Park,[1] Il-Buem Lee,[1] Hyeon-Min Moon,[1] Seok-Cheol Hong,[1,2]* Minhaeng Cho[1,3]*

[1]Center for Molecular Spectroscopy and Dynamics, Institute for Basic Science, Seoul 02841, Korea

[2]Department of Physics, Korea University, Seoul 02841, Korea

[3]Department of Chemistry, Korea University, Seoul 02841, Korea

*Corresponding author. Email: hongsc@korea.ac.kr, mcho@korea.ac.kr.

Contents

Supplementary Fig. 1. Experimental design of fluorescence-combined iSCAT microscopy setup.

Supplementary Fig. 2. Two image processing methods used to enhance the contrast of the iSCAT image and to identify dynamic cargos.

Supplementary Fig. 3. Continuous cargo tracking with SBR- and TD-iSCAT methods.

Supplementary Fig. 4 Estimation of the precision of localization of a dynamic cargo.

Supplementary Fig. 5. Size distribution of cargos in SBR-iSCAT snapshots.

Supplementary Fig. 6. Cytoskeletal highways reconstructed by cargo localization and kinetics of intracellular cargos.

Supplementary Fig. 7. Measurement of the persistence length (ξ) of microtubule through curvature analysis.

Supplementary Fig. 8. Instantaneous speed of a moving cargo measured with three different time windows.

Supplementary Fig. 9. Cargo packets.

Supplementary Fig. 10. Propagation of packets of cargos to the site of a growing filopodium and their discharging thereafter.

Supplementary Fig. 11. Intracellular transport network of a COS-7 cell captured by F-iSCAT imaging.

Supplementary Fig. 12. Impact of nocodazole treatment on intracellular traffic in a COS-7 cell.

Supplementary Fig. 13. Imaging of GFP-labeled late endosomes (LEs) in a COS-7 cell using F-iSCAT microscopy.

Supplementary Fig. 14. Detection and counting of cargos in a traffic-jammed area.

Supplementary Fig. 15. Long journey of dimeric cargos observed by iSCAT microscopy.

Supplementary Fig. 16. Dissociation of a dimeric cargo.

Supplementary Fig. 17. Directional transport of a trimeric cargo and its dissociation.

Supplementary Fig. 18. Dynamic events revealed by a dimeric cargo via direct interactions with the cellular environment.

Supplementary Fig. 19. U-turn of a dimeric cargo.

Supplementary Fig. 20. Cargo identification in TD-iSCAT images based on the parameter of intensity percentile (I_p) in the MOSAIC ImageJ plugin.

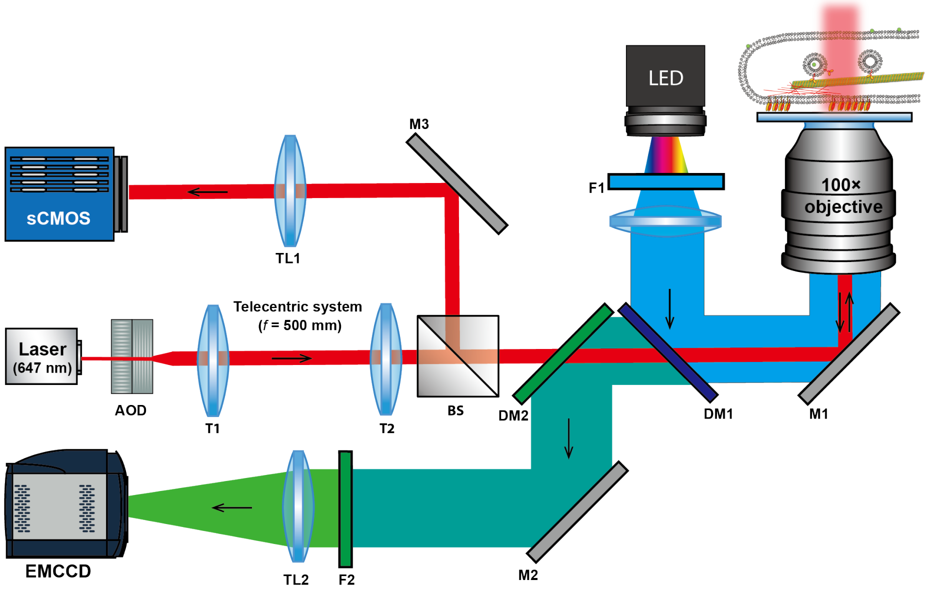

Supplementary Fig. 1. Experimental design of fluorescence-combined iSCAT microscopy setup. A 647-nm laser beam (red), which is the light source for iSCAT imaging, is steered by a two-axis AOD and then collimated by a set of telecentric lenses (T1 and T2, f = 500 mm). The reflected and scattered lights are collected back through the objective lens, split with a 50R:50T beam splitter (BS), and imaged onto the sCMOS camera via a tube lens (TL1, f = 750 mm). A broad spectrum of white LED light, which is the light source for fluorescence microscopy, is filtered through the bandpass filter (F1) to blue light suitable to excite f-PS beads. The fluorescent signals from f-PS beads are projected onto the EMCCD camera via a tube lens (TL2, f = 400 mm) after passing through the emission filter (F2) chosen to select the fluorescent light from f-PS beads. Here, two dichroic mirrors (DM1 and DM2) are used to reflect the excitation beam into the main beam path and the fluorescent signal into the detector. Each arrow indicates the direction of beam propagation.

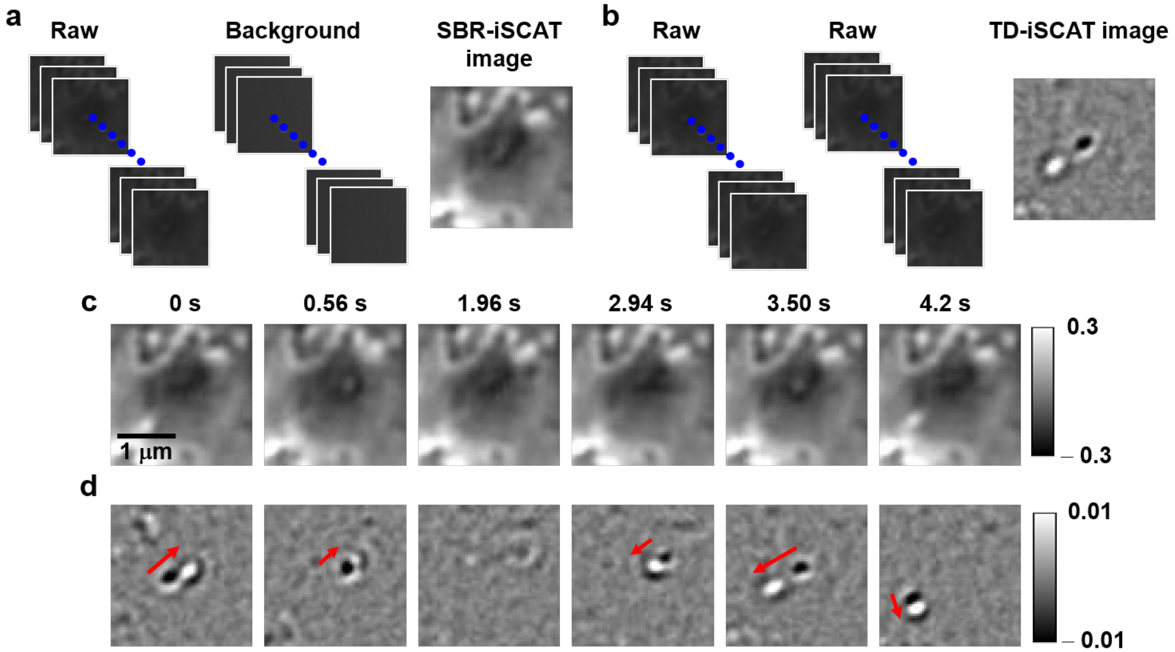

Supplementary Fig. 2. Two image processing methods used to enhance the contrast of the iSCAT image and to identify dynamic cargos. **a,** SBR-iSCAT image generated by dividing a temporal-median cell image by the relevant temporal-median background image. A temporal-median cell image at t is obtained by averaging 10 consecutive frames (from $t - \Delta t$ to t with $\Delta t = 0.2$ s) of raw image. A temporal-median background image is taken from a cell-free area, providing time-invariant background signals. The background image at t is obtained by averaging 3000 consecutive frames (from $t - \Delta t$ to t with $\Delta t = 60$ s) of raw image. **b,** TD-iSCAT image generated by dividing a temporal-median cell image by another temporal-median cell image taken from the same area at the time interval of 0.2 s. In TD-iSCAT imaging, a temporal-median cell image is averaged over 20 consecutive frames of raw image ($\Delta t = 0.4$ s). One moving cargo is clearly visible in the form of a pair of bright and dark spots. **c, d,** Sequences of SBR-iSCAT (c) and TD-iSCAT images (d) that capture the cellular landscape including a moving cargo and the moving cargo only, respectively. In the TD-iSCAT image, the dark and bright spots correspond to the initial and final positions of the cargo during each observation time span ($\Delta t = 0.4$ s). In (d), the direction and length of arrows represents the displacement this cargo has for Δt, which correlates with its velocity. When a cargo stops, it disappears in the TD-iSCAT image at $t = 1.96$ s. Grayscale legends in (c) and (d) are used as in Fig. 1c and 1d, respectively.

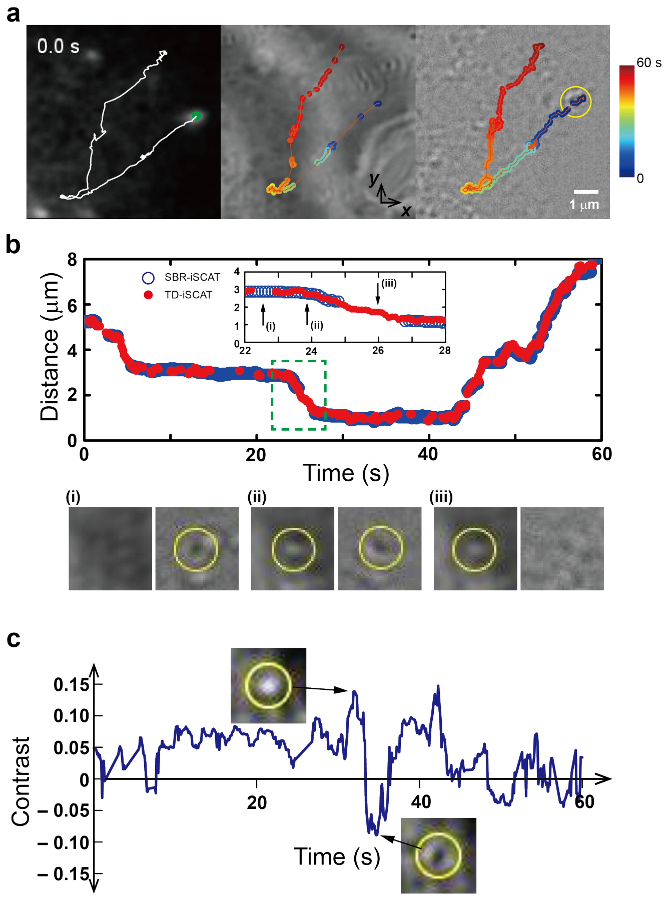

Supplementary Fig. 3. Continuous cargo tracking with SBR- and TD-iSCAT methods. **a,** Trajectories of a GFP-labeled late endosomal cargo, revealed by fluorescence detection (left, white) and SBR-iSCAT (middle) or TD-iSCAT (right) imaging-based cargo localization. The trajectories by iSCAT are color-coded based on the elapsed time and superimposed on a snapshot taken at t = 0.0 s. Color legend is used as in Fig. 1g. **b,** Comparison of the two trajectories of the cargo obtained by SBR- and TD-iSCAT methods (blue: SBR-iSCAT; red: TD-iSCAT) highlighting the complementarity of the two methods for persistent cargo tracking. Only the y-positions of the cargo from the trajectories are plotted for ease of comparison. Three representative moments when the cargo was detected by TD-iSCAT only, SBR-iSCAT only, or both, marked as (i), (iii), and (ii) respectively, are captured by snapshots (left: SBR-iSCAT; right: TD-iSCAT; yellow circle: detected cargo). The trace of the cargo with SBR-iSCAT was successfully acquired except for a few moments where it was masked by an unknown object (case (i)). The cargo was frequently lost in TD-iSCAT due to its intermittent pauses (case (iii)). **c,** The SBR-iSCAT contrast of the cargo, reflecting its axial motion, is shown with images of the cargo in the brightest and darkest contrast next to a peak and a trough in the time trace, respectively. See also Supplementary Movie 3. Source data are provided in a Source Data file.

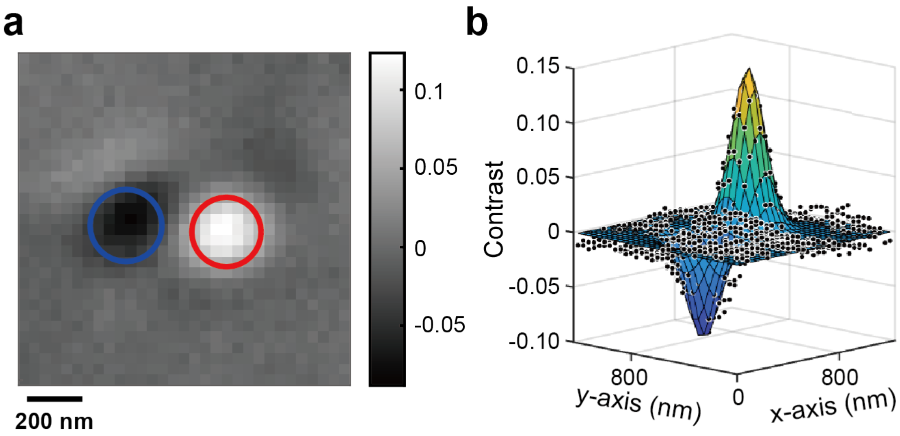

Supplementary Fig. 4. Estimation of the precision of localization of a dynamic cargo. **a,** A dynamic cargo appears as a pair of bright (red circle) and dark (blue circle) spots in a TD-iSCAT snapshot. Grayscale legend is used as in Fig. 1d. **b,** Contrast image (surface plot) of a pair of bright and dark spots shown in (a) and its 2D Gaussian fit. The values of SNR for the bright and dark spots were estimated to be ~ 6 and ~ 4, respectively. The estimated precisions of localization of the bright and dark spots were then about 10 and 15 nm, respectively.

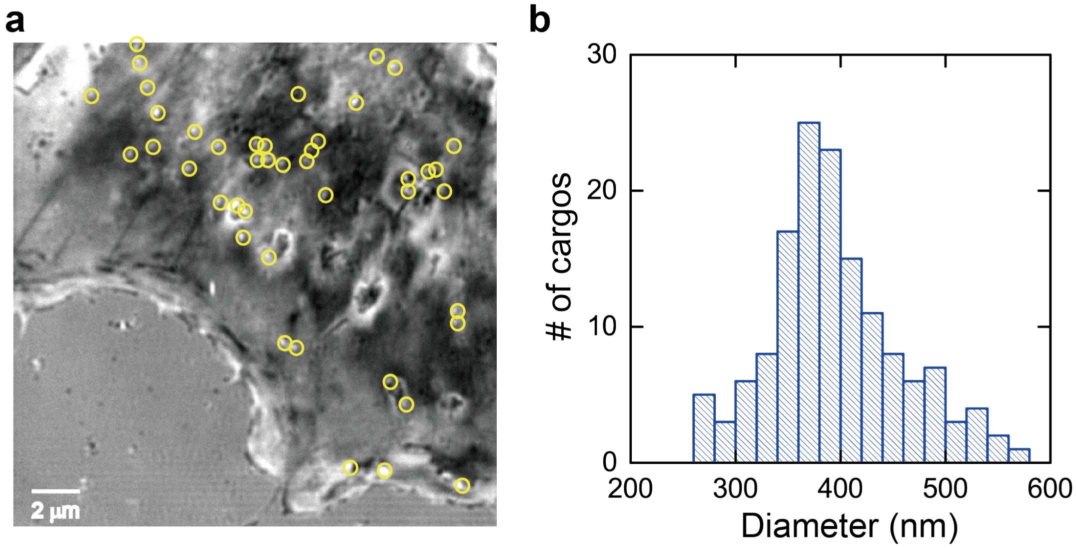

Supplementary Fig. 5. Size distribution of cargos in SBR-iSCAT snapshots. **a,** Cargos (yellow circles) identified in a SBR-iSCAT snapshot at $t = 0$ using the MOSAIC ImageJ plugin with the particle radius (r) of 7, cutoff score (s_{cut}) of 0, and intensity percentile (I_p) of 0.5. **b,** Histogram of cargo size for 144 cargos detected from three SBR-iSCAT snapshots taken at $t = 0$, 20, and 40 s. The average size measured was 393 ± 62 nm. Source data are provided in a Source Data file.

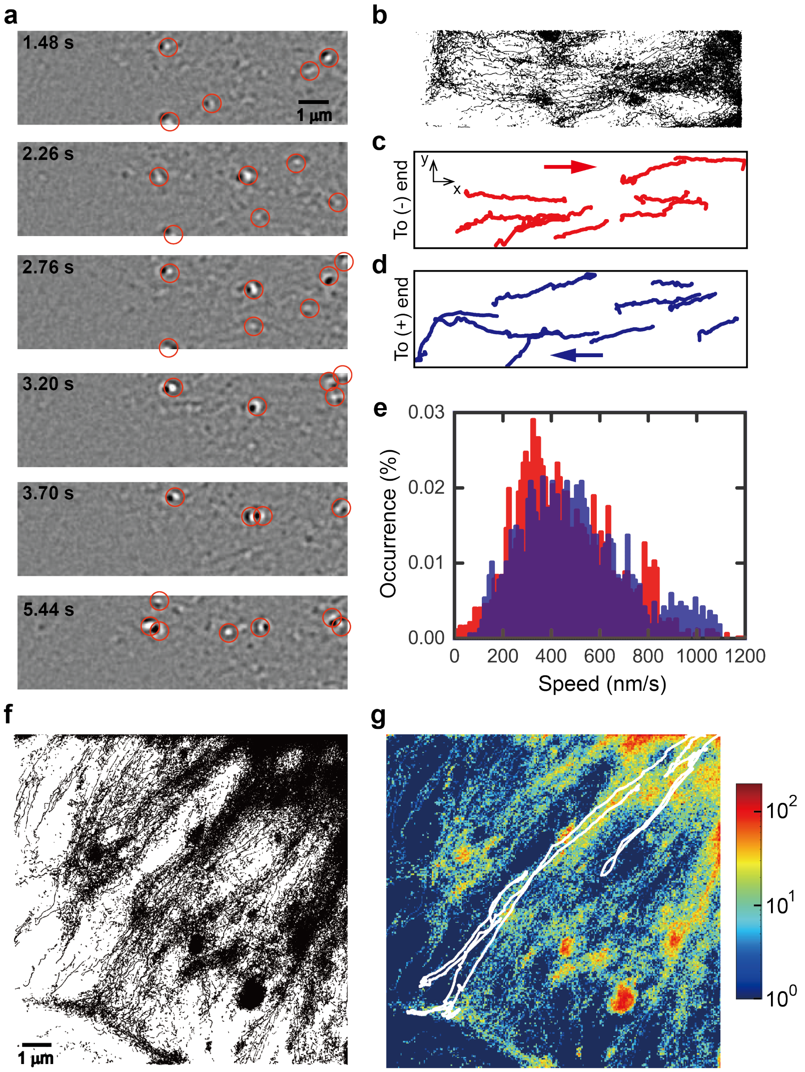

Supplementary Fig. 6. Cytoskeletal highways reconstructed by cargo localization and kinetics of intracellular cargos. **a,** Sequence of TD-iSCAT images overlaid with cargo localization positions (circles), taken from the white box drawn in Fig. 1d. **b,** Accumulation of about one hundred thousand cargo positions identified in consecutive 50,000 images ($\Delta T \sim 17$ min) in the same area as in (a). **c, d,** 9 and 10 trajectories generated by cargos transported to the cell body ((−) end) (c) and boundary ((+) end) (d), respectively, selected from (b). **e,** Histogram showing the instantaneous speed of cargos moving to the (−) and (+) ends in (e) (red and blue, respectively). **f, g,** Reconstituted cytoskeletal network (f) and traffic density map (g) (blue-boxed area in Fig. 1c). Color legend is used as in Fig. 2c except for using 50,000 frames. More than half a million points from 5000 consecutive frames taken at 50 Hz were used to make this map. One representative trajectory of an f-PS bead (white line) was overlaid on the traffic density map in (g). Source data are provided in a Source Data file.

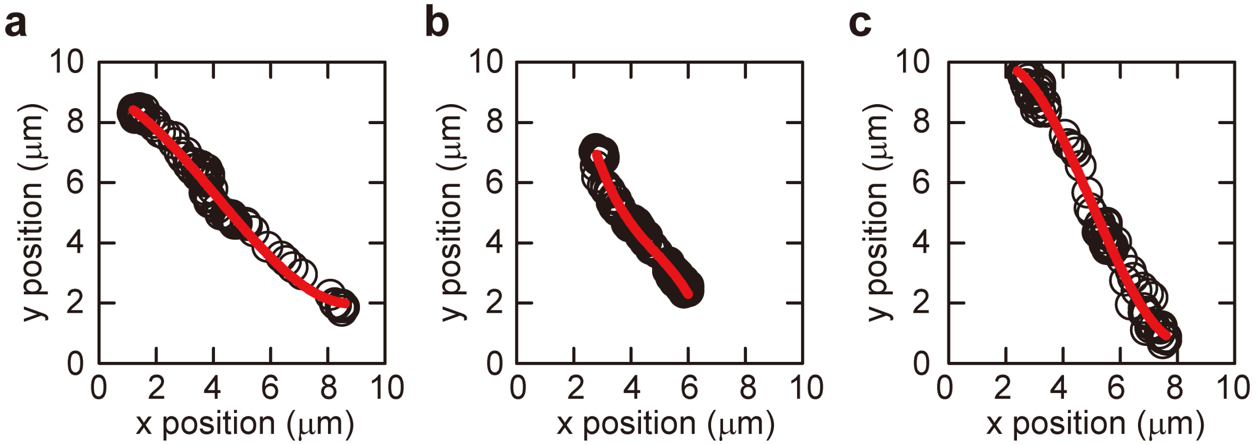

Supplementary Fig. 7. Measurement of the persistence length (ξ) of microtubule through curvature analysis of the shape of a microtubule reconstructed from cargo positions. Three representative trajectories of microtubule were obtained by continuously tracking cargos embedded with an f-PS bead using fluorescence microscopy at a frame rate of 10 Hz (a-c). Raw data points (empty circles) in (a-c) delineate the underlying microtubule's structures. The values of ξ were calculated to be 234, 246, and 289 μm in (a-c), respectively, by analyzing the fitted curves (red) derived from the raw date points using the open-source code provided by Wisanpitayakorn et al[30]. Source data are provided in a Source Data file.

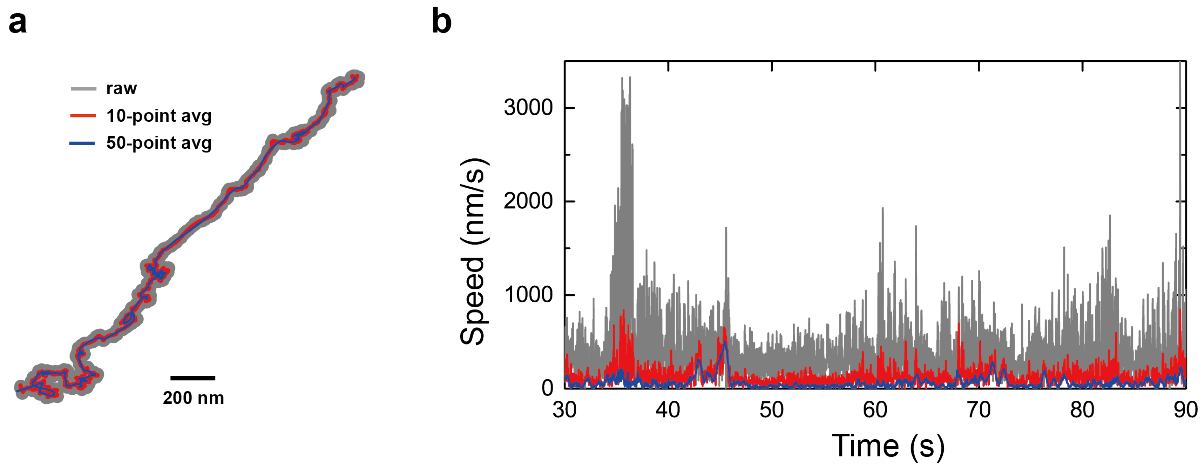

Supplementary Fig. 8. Instantaneous speed of a moving cargo measured with three different time windows. **a,** A typical raw (gray) trajectory of a cargo overlaid with 10-point (red; Δt = 0.2 s) and 50-point (blue; Δt = 1 s) time-averaged trajectories (Fig. 3e). The MOSAIC ImageJ plugin was used to analyze raw TD-iSCAT images and track the cargo automatically. See also Supplementary Movie 7. **b,** Instantaneous speed calculated from the raw (gray), 10-point averaged (red), and 50-point averaged (blue) trajectories in (a). Source data are provided in a Source Data file.

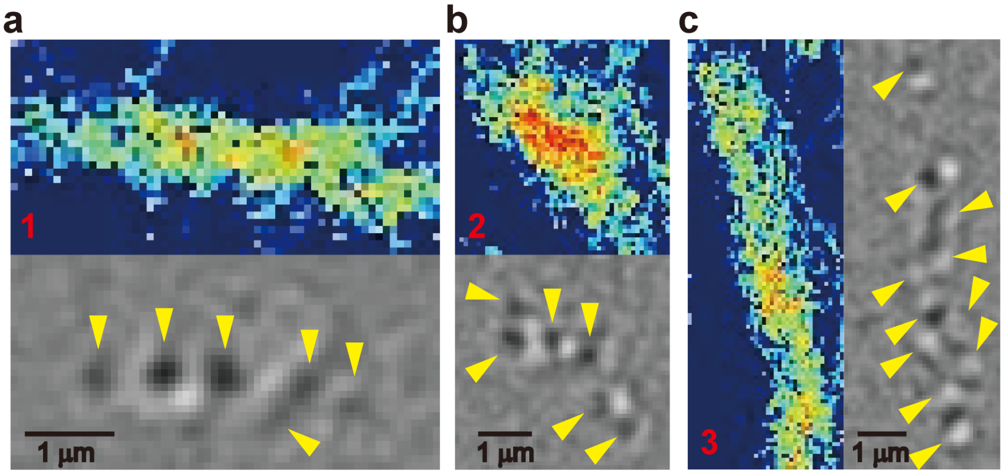

Supplementary Fig. 9. Cargo packets. Densely packed clusters of cargos that move collectively, marked by red dotted ovals (1, 2, and 3) in Fig. 2d. Individual cargos within the packets are indicated by yellow arrowheads in TD-iSCAT snapshots taken at t = 361.8 s (a), 410.0 s (b), and 568.7 s (c).

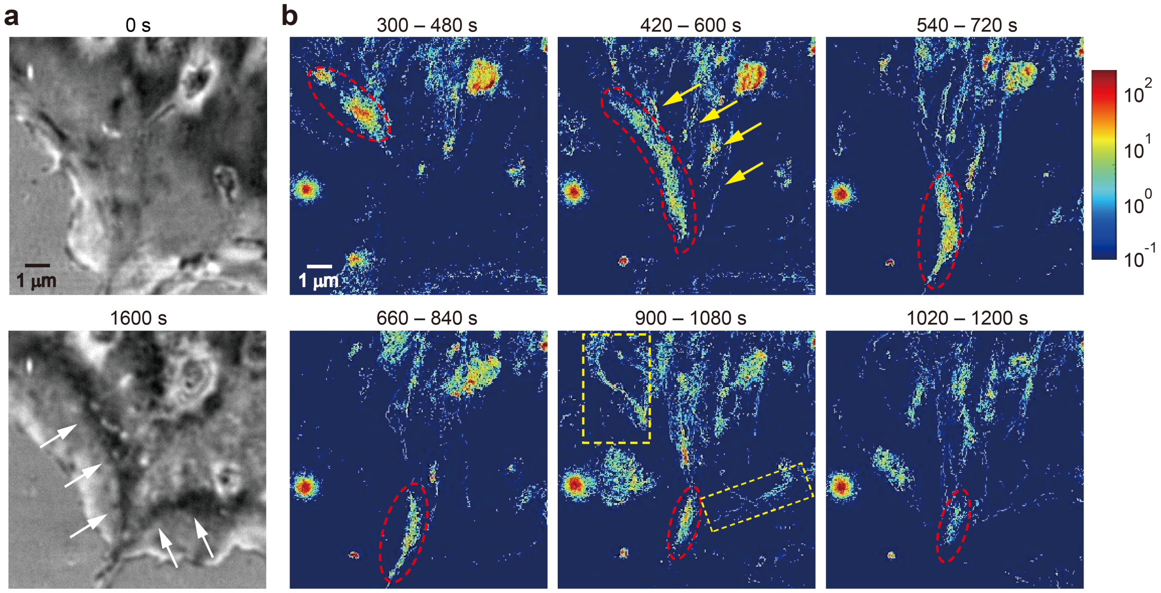

Supplementary Fig. 10. Propagation of packets of cargos to the site of a growing filopodium and their discharging thereafter. **a,** Two SBR-iSCAT images taken at t = 0 and 1600 s (purple-boxed area in Fig. 2a). The morphology of the cell changed at the cell boundary over 1600 s. **b,** Sequence of cargo-localization density maps integrated for ΔT = 180 s. The packet of cargos (red oval) rushes to a protruding area at the boundary (1st to 4th image). During the process, the traffic density soars as other packets (yellow arrows) are combined at the intersection of cytoskeleton highways. Once it reached the protruding area, it disappeared (5th and 6th image). Other packets emerged, as indicated by yellow boxes in the 5th image. Color legend is used as in Fig. 2d. Source data are provided in a Source Data file.

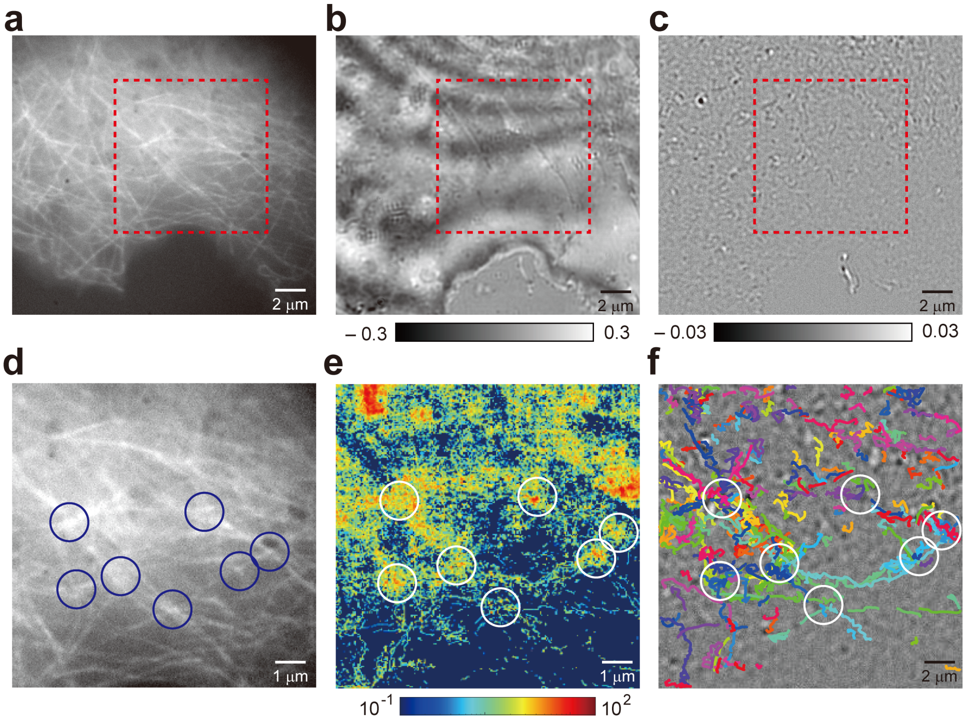

Supplementary Fig. 11. Intracellular transport network of a COS-7 cell captured by F-iSCAT imaging. **a,** Fluorescence image displaying the spatial distribution of microtubules labeled with GFP. **b,** SBR-iSCAT and **c,** TD-iSCAT snapshots corresponding to the fluorescence image. Grayscale legends in (b) and (c) are used as in Fig. 1c and 1d, respectively. **d,** Magnified fluorescence image of the red-boxed area in (a). This area highlights seven microtubule intersections, marked by blue circles. **e,** The cargo-localization density map was created from 15,000 consecutive iSCAT images taken at 50 Hz, showing microtubule intersections as concentrated areas of cargo (white circles). Here, color legend is used as in Fig. 2c except for using 15,000 frames (ΔT = 300 s). **f,** 402 trajectories of individual cargos superimposed onto the magnified TD-iSCAT image of the red-boxed area in (c) display the active transport routes through the microtubule network. The white circles in (e) and (f) that correspond to the microtubule intersections marked by blue circles in (d) also exhibit high density regions in the density map in (e) and serve as primary passageways for cargo transportation where multiple cargo trajectories intersect with each other as shown in (f). The cargos selected here are among those tracked continuously over 1 s. Source data are provided in a Source Data file.

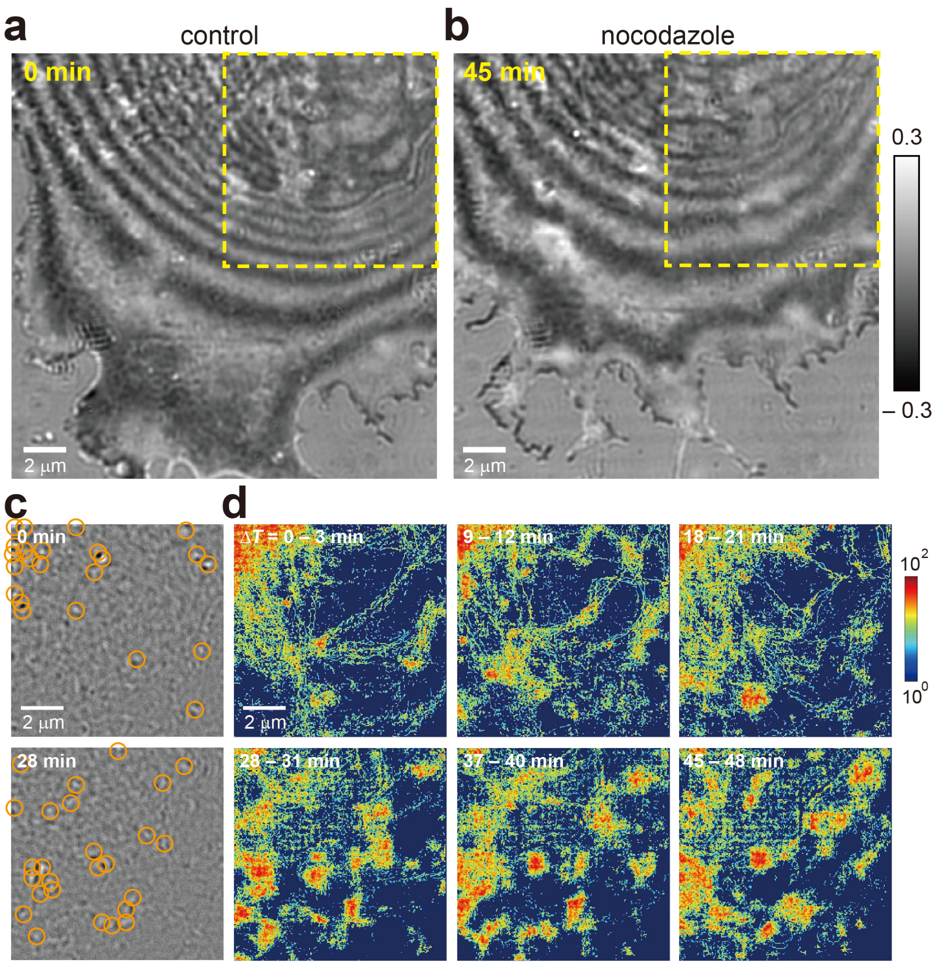

Supplementary Fig. 12. Impact of nocodazole treatment on intracellular traffic in a COS-7 cell. **a,** SBR-iSCAT snapshots of a COS-7 cell before and **b,** after treatment with 2 μM nocodazole. Nocodazole was treated at t = 20 min. Grayscale legend is used as in Fig. 1d. **c,** Locations of dynamic cargos (orange circles) identified from TD-iSCAT snapshots (yellow-boxed area in (a, b)) taken at two different time point, t = 0 and 28 min. **d,** Sequential images of cargo localization density map of the yellow-boxed area in (a, b). Each image was generated from 9,000 consecutive frames taken at 50 Hz. Supplementary Movie 6 and 7 show cargo dynamics before and after nocodazole treatment, respectively. In (d), color legend is used as in Fig. 2d. Source data are provided in a Source Data file.

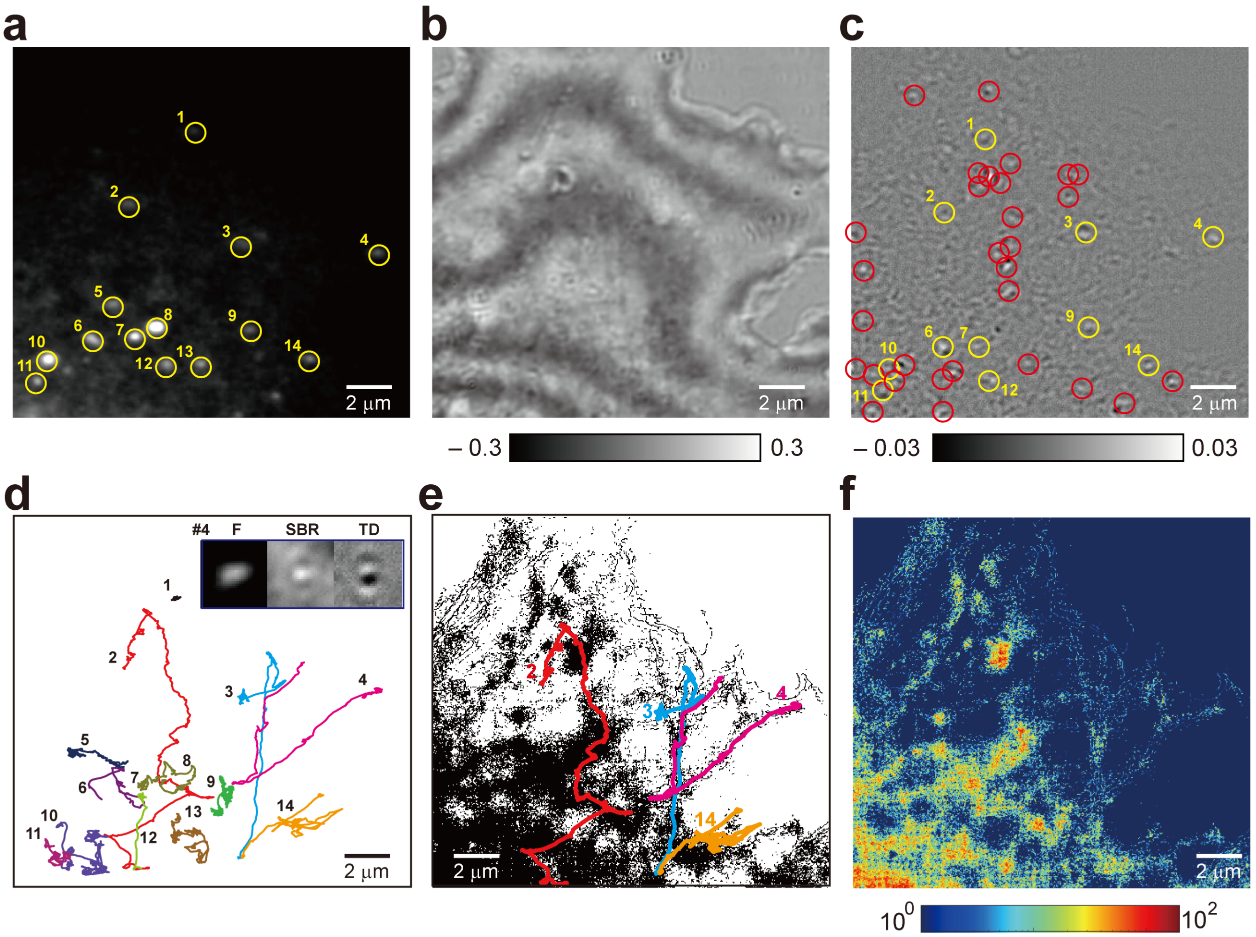

Supplementary Fig. 13. Imaging of GFP-labeled late endosomes (LEs) in a COS-7 cell using F-iSCAT microscopy. **a,** Fluorescence image showing 14 LEs marked by numbered yellow circles. **b,** SBR-iSCAT and **c,** TD-iSCAT images corresponding to (a). Yellow circles with the same numbers in (a) indicate co-localized cargos, *i.e.*, LEs detected in TD-iSCAT image, while red circles indicate other cargos only detected in TD-iSCAT image. Grayscale legends in (b) and (c) are used as in Fig. 1c and 1d, respectively. **d,** Trajectories of 14 LEs numbered as in (a) and tracked at 10 Hz using fluorescence microscopy, depicted in different colors. (Inset: typical snapshots of LE (#4) captured by fluorescence (F), SBR- and TD-iSCAT images.) **e,** Cytoskeleton network reconstructed from TD-iSCAT images using cargo localization, based on 15,000 consecutive frames taken at 50 Hz. Four representative trajectories of LEs (#2, #3, #4, and #14) from (d) are overlaid. **f,** Traffic density map showing the total number of cargos detected at each pixel for a total of 15,000 consecutive frames (ΔT = 300 s), indicated by the color legend. See also Supplementary Movie 3. Source data are provided in a Source Data file.

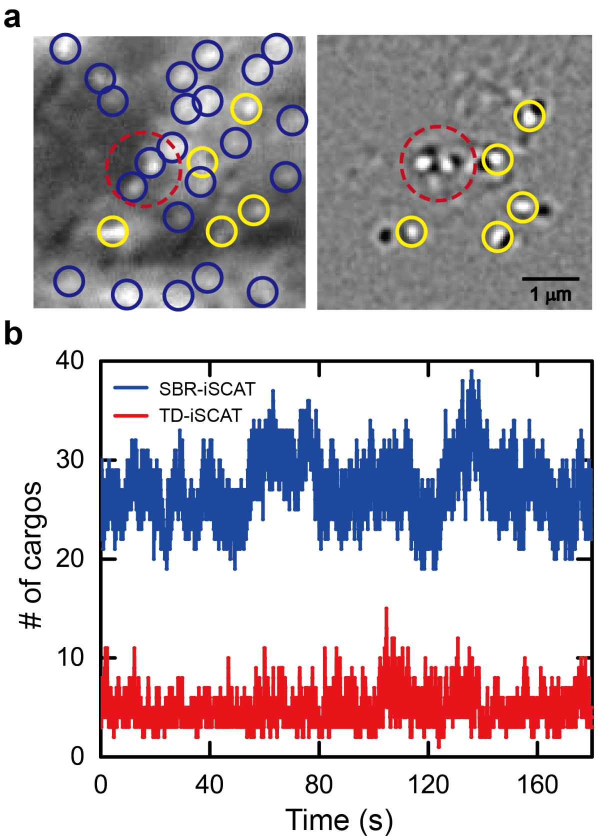

Supplementary Fig. 14. Detection and counting of cargos in a traffic-jammed area, also shown in Fig. 3a, by SBR- and TD-iSCAT methods. **a,** Cargos (represented as circles) in snapshots of SBR-iSCAT (left) and TD-iSCAT (right) detected by the MOSAIC ImageJ plugin. Circles in yellow represent cargos detected simultaneously in both snapshots while those in blue were only detected in SBR-iSCAT. The red-dashed circle highlights a cargo-jamming spot in the TD-iSCAT image, where at least 3 cargos (blue circles in red-dashed circle) were identified in the complementary SBR-iSCAT image. **b,** Temporal variation in the number of cargos in the area shown in (a), as detected by SBR-iSCAT (blue) and TD-iSCAT (red). Image acquisition rate: 50 Hz. Source data are provided in a Source Data file.

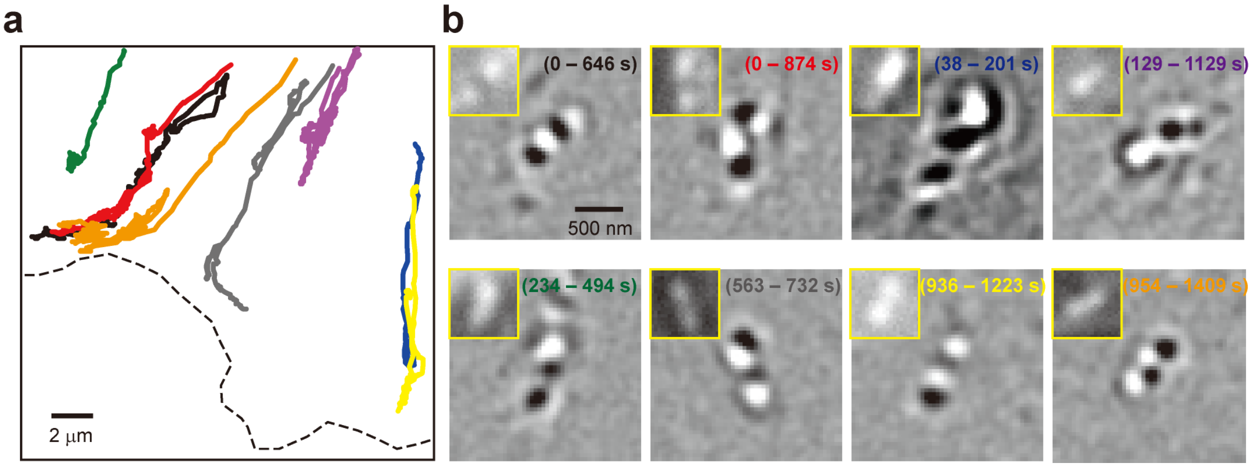

Supplementary Fig. 15. Long journey of dimeric cargos observed by iSCAT microscopy. **a,** Trajectories of eight dimeric cargos tracked within a single lamellipodial region, as shown in Fig. 1f. The dotted line indicates the cell boundary. **b,** Snapshots of the eight dimeric cargos captured by TD-iSCAT imaging. The colored number in parenthesis indicates the period of the successful cargo tracking for each dimer. The same color is used to represent the same dimeric cargo in both (a) and (b). The insets in each panel of (b) show the corresponding SBR-iSCAT images, which depict the shape of the dimeric cargo. Source data are provided in a Source Data file.

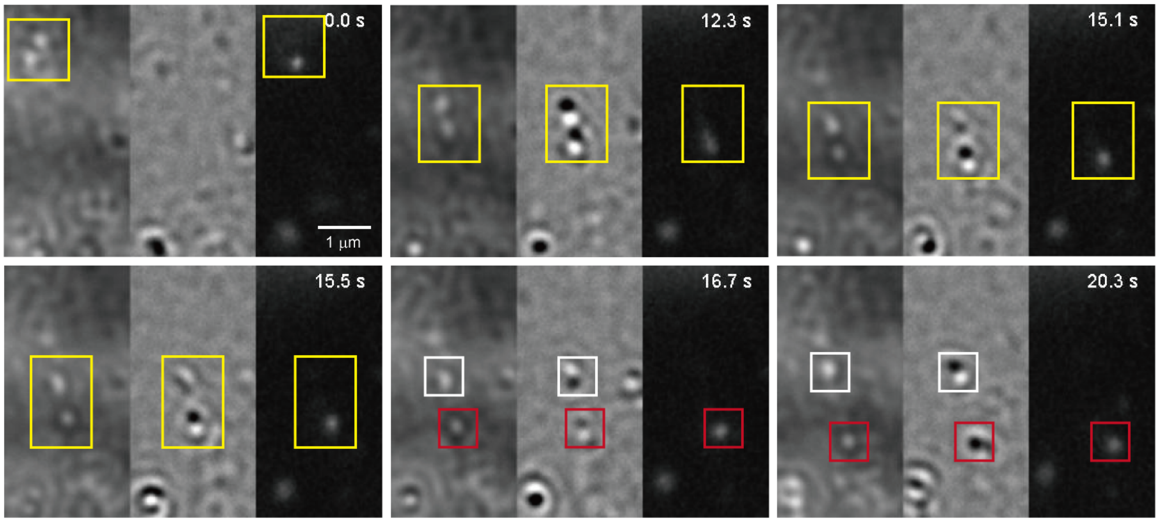

Supplementary Fig. 16. Dissociation of a dimeric cargo. The separation of a dimeric cargo is depicted in boxed areas in SBR-iSCAT (left), TD-iSCAT (middle), and fluorescence (right) images taken at six time points. At $t \sim 15$ s, the front cargo (red box) moves forward continuously while the rear cargo (white box) stalls. The front cargo contains a f-PS bead, as indicated by fluorescence. See also Supplementary Movie 11.

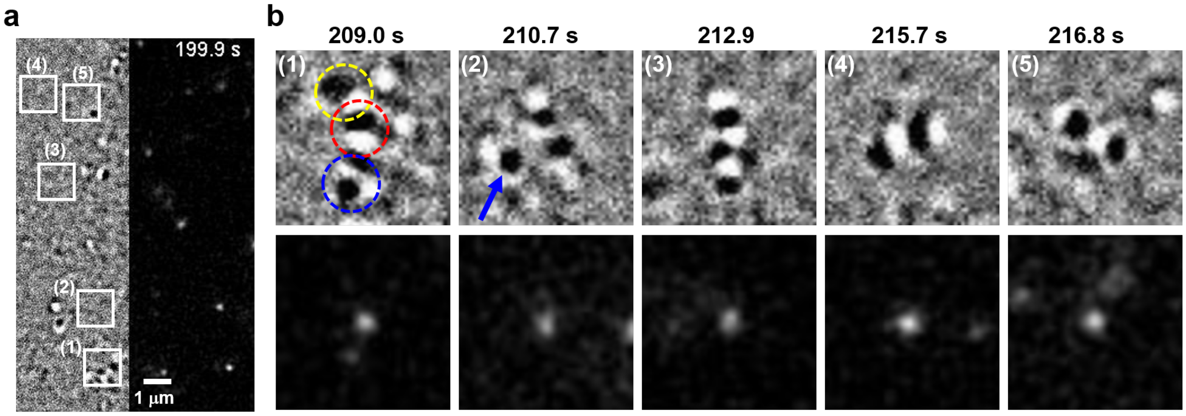

Supplementary Fig. 17. Directional transport of a cargo trimer and its dissociation. **a,** iSCAT snapshot showing intracellular cargos in a live COS-7 cell (left) and its complementary fluorescence image showing the locations of f-PS beads internalized into the cell. **b,** Sequential iSCAT images (numbered from (1) to (5)) showing a cargo trimer moving together along a one-dimensional track (yellow, red, and blue circles in the 1st image of the upper sequence). Among them, the cargo at the bottom of the image (blue circle) at t = 209.0 s was detached at t = 210.7 s, as indicated by the blue arrow. On the other hand, the other two cargos moved together as a dimer and turned around at the intersection of cytoskeletons (t = 215.7 and 216.8 s). The areas viewed in the sequence of images (from image (1) to (5)) are marked by white boxes with the same number in the left image of (a). As shown in the fluorescence images (lower panels), one cargo module in the dimer contained an f-PS bead. See also Supplementary Movie 12.

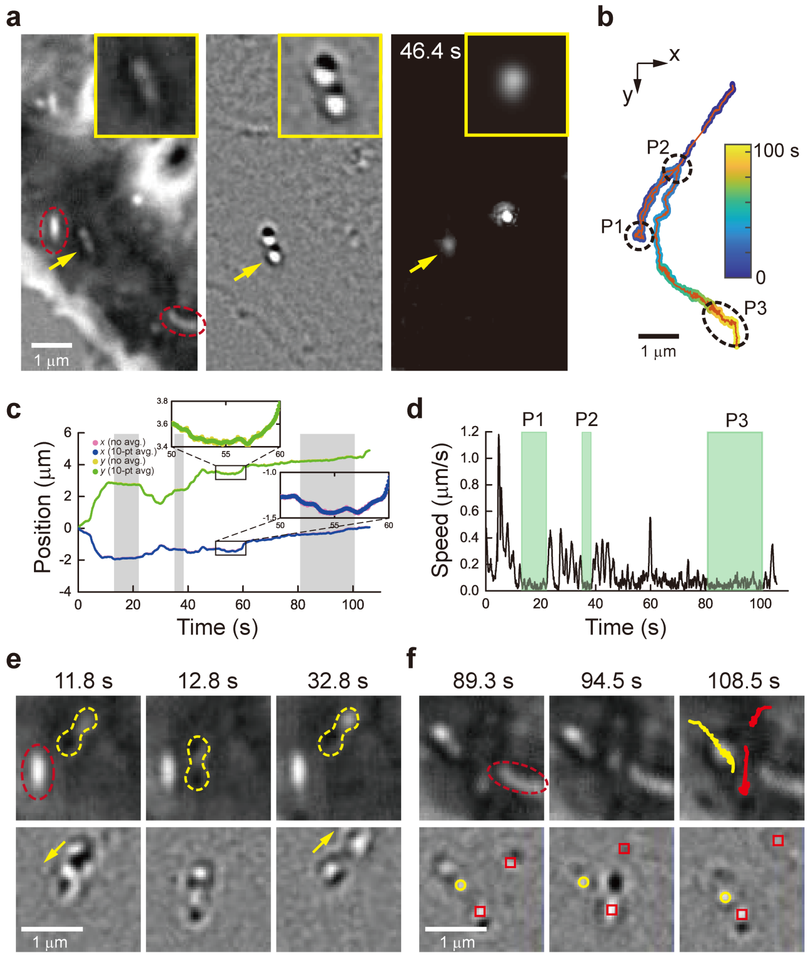

Supplementary Fig. 18. Dynamic events revealed by a dimeric cargo via direct interactions with the cellular environment. **a,** Identification of a dimeric cargo by dual-channel observation of the cargo with fluorescence-combined iSCAT microscopy in the red-boxed area in Fig. 1c (left: SBR-iSCAT; middle: TD-iSCAT; and right: fluorescence). An unlabeled cargo moves together with another cargo bearing an f-PS bead throughout our observation. **b,** Trajectory of the f-PS bead tracked for ~ 100 s at 10 Hz using fluorescent microscopy. Color legend is used as in Fig. 1g. **c,** 10-point time-averaged traces of *x* and *y* positions of the bead in (b). Insets show the scatter of raw *x* and *y* positions over time. **d,** 'Instantaneous speed' of the f-PS bead calculated from 10-point time-averaged data points with Δt = 0.1 s. Three pausing events observed during cargo transport are highlighted by green shades (P1, P2, and P3), corresponding to three circled incidents drawn on the trajectory in (b). **e,** Halted cargo transport at P1 due to direct contact with a cytoplasmic obstacle (red oval marked in the 1st image). In the SBR-iSCAT images, the location of the dimeric cargo is outlined by yellow-dashed lines. The arrows in TD-iSCAT images indicate the direction of cargo movement. Around the obstacle, the cargo roved in place. **f,** Delayed cargo transport at P2 due to traffic jamming. Two other cargos, indicated by red boxes, were observed to cross the junction ahead of time, interrupting the movement of the dimeric cargo. There must be an intersection of two perpendicular cytoskeletal tracks at P2. The trajectories of those cargos are overlaid in the 3rd of the SBR iSCAT images. Finally, it was paused again near the edge of the lamellipodium (P3) as shown in (b). See also Supplementary Movie 13. Source data are provided in a Source Data file.

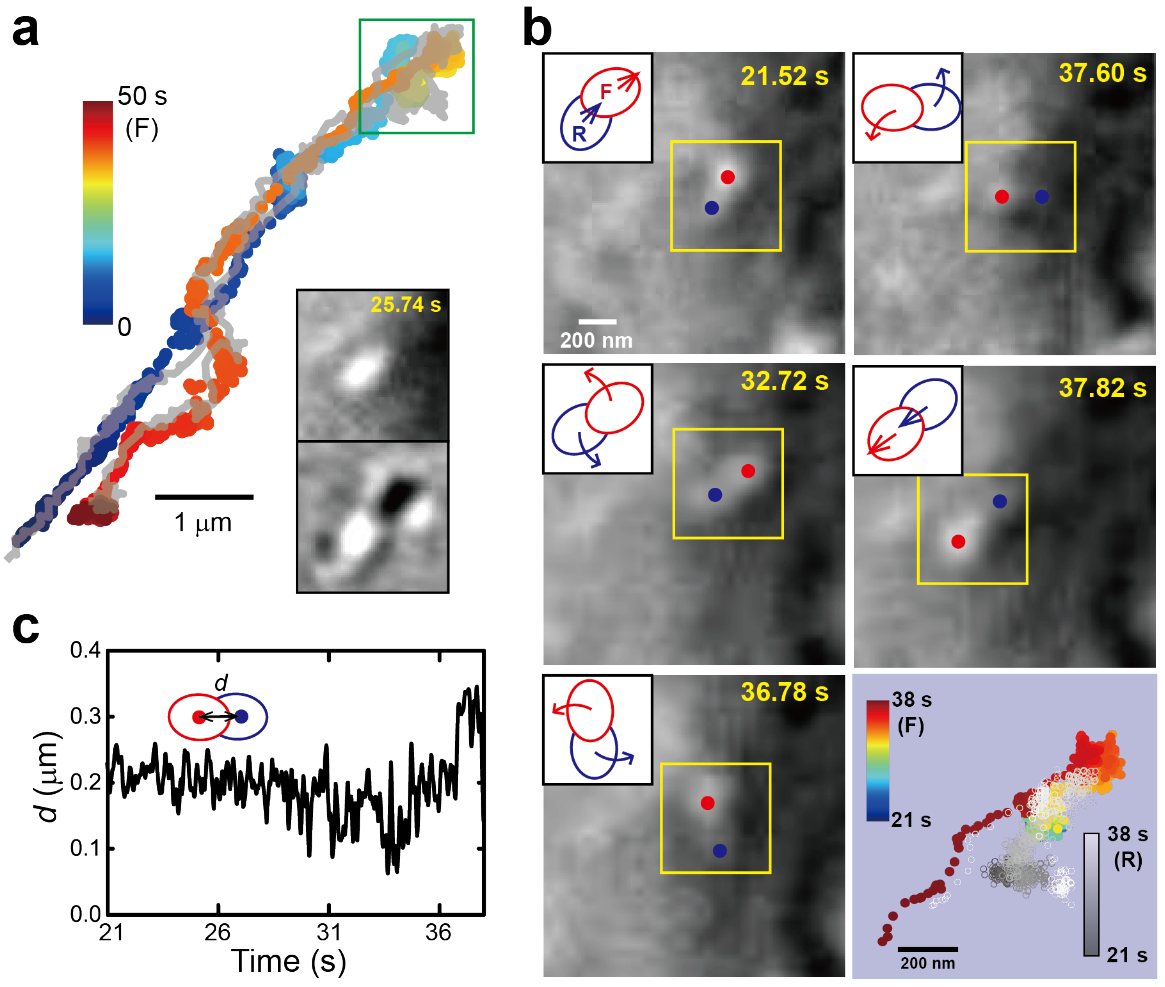

Supplementary Fig. 19. U-turn of a dimeric cargo. **a,** Trajectories of the two cargos moving in a dimeric form (front cargo: color-coded by elapsed time (see color legend); rear cargo: gray). Insets: Snapshots of the dimeric cargo taken at t = 25.74 s using SBR-iSCAT (upper) and TD-iSCAT (lower) imaging. **b,** Sequence of images taken at five time points showing the U-turn of the dimeric cargo (red dot: front cargo; blue dot: rear cargo) and the zoomed-in trajectories in the green-boxed area in (a). Color and grayscale legends represent time points for front and rear cargos, respectively. The direction of motion of the front and rear cargos is indicated by red and blue arrows, respectively. **c,** The distance (d, marked by arrow) between the front and rear cargos remained less than 300 nm during observation. The center location of each cargo was calculated by fitting with a 2D Gaussian function. See also Supplementary Movie. 14. Source data are provided in a Source Data file.

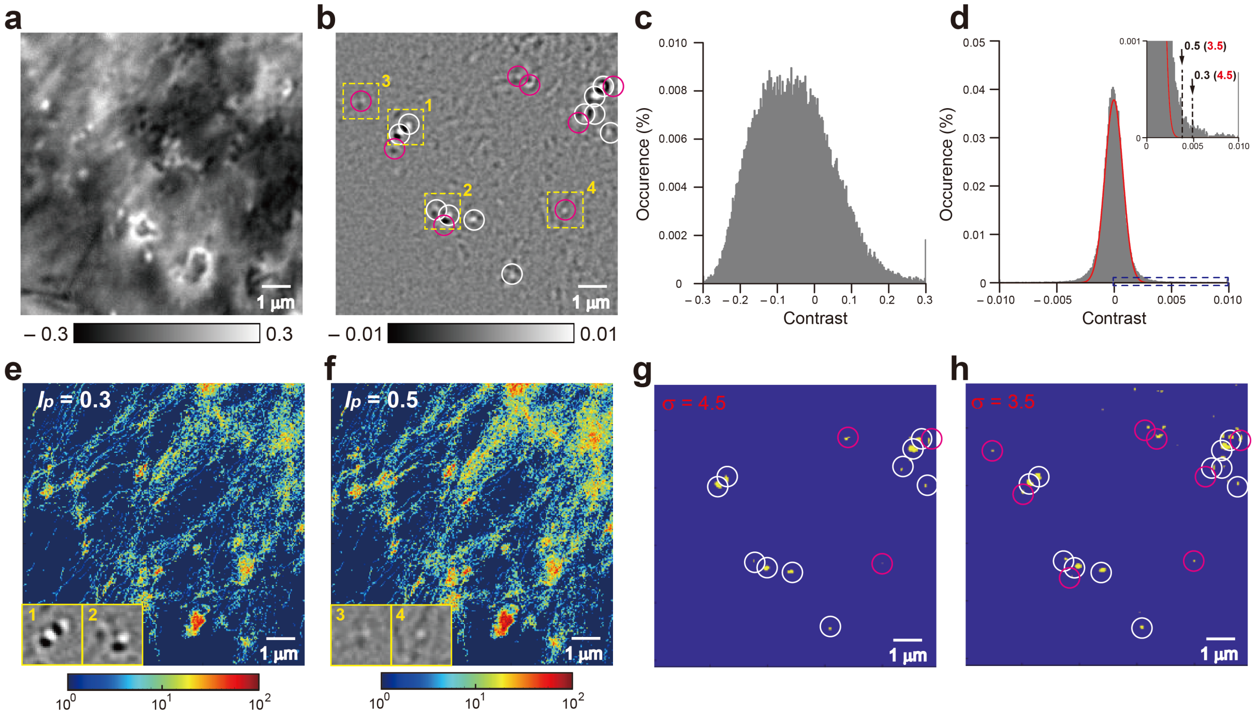

Supplementary Fig. 20. Cargo identification in TD-iSCAT images based on the parameter of intensity percentile (I_p) in the MOSAIC ImageJ plugin. **a, b,** SBR-iSCAT image (a) and its corresponding TD-iSCAT image (b). In (b), cargos were identified with I_p = 0.5. Cargos with I_p = 0.3 were marked by white circles. Cargos, marked by pink circles, were those detected additionally with I_p = 0.5. **c, d,** Histograms showing the distribution of iSCAT contrast taken from the whole pixels in the SBR- (a) and TD-iSCAT images (b). In the inset of (d), two dotted lines indicate the locations corresponding to the threshold values, I_p = 0.5 and 0.3. The thresholds set by I_p = 0.5 and 0.3 are nearly equivalent to those set by μ + 3.5σ and μ + 4.5σ, respectively, with the mean, μ, and the standard deviation, σ, obtained from Gaussian fitting (red) to the histogram. **e, f**, Cargo-localization density maps reconstructed from 9,000 consecutive TD-iSCAT images using two different parameter values of I_p = 0.3 (e) and 0.5 (f). The insets in (e) and (f) represent the TD-iSCAT images of cargos detected with I_p = 0.3 and in the percentile interval from 0.3 to 0.5, respectively. A proximal pair of bright and dark spots, typical feature of a dynamic cargo detected with I_p = 0.3, is clearly visible in the TD-iSCAT image. However, with I_p = 0.5, spots with the bright contrast only were additionally detected, indicating that setting a lower threshold likely result in false positive counts. **g, h,** Bright spots (yellow) identified in the TD-iSCAT image in (b) by the level of intensity (μ + 4.5σ and μ + 3.5σ), which correspond to I_p = 0.3 and 0.5, respectively. White and pink circles in (g, h) indicate the locations of bright spots identified as cargos in the TD-iSCAT image with I_p = 0.3 and 0.5, respectively. Other spots, not detected as cargos in the TD-iSCAT image in (b), were found in the threshold of μ + 3.5σ. The SBR-iSCAT image in (a) corresponds to the blue-boxed area in Fig. 1c. Source data are provided in a Source Data file.
