# Supplementary material for: Long-term cargo tracking reveals intricate trafficking through active cytoskeletal networks in the crowded cellular environment
Source: Nat Commun. 2023 Nov 14;14:7160. doi: 10.1038/s41467-023-42347-7 (PMC10645962; doi:10.1038/s41467-023-42347-7)
Supplement: Supplementary file 3 — Description of Additional Supplementary Files [file 41467_2023_42347_MOESM3_ESM.pdf]

Description of Additional Supplementary Files

Supplementary Movie 1. Directional f-PS bead in a live COS-7 cell. An f-PS bead displaying the bi-directional transport in a live COS-7 cell was captured in a time-lapse fluorescent microscopy movie taken at 10 Hz. This f-PS bead was also simultaneously tracked by iSCAT, as shown in the upper inset of Fig. 1g. Scale bar: 1 μm.

Supplementary Movie 2. Brownian f-PS bead on a live COS-7 cell. A Brownian f-PS bead on the membrane surface of a live COS-7 cell was captured in a time-lapse fluorescent microscopy movie taken at 10 Hz. This f-PS bead was not detected by iSCAT, as shown in the lower inset of Fig. 1g. Scale bar: 200 nm.

Supplementary Movie 3. Continuous tracking of a GFP-labeled late endosomal cargo in a live COS-7 cell with F-iSCAT microscopy. A GFP-labeled late endosomal cargo was tracked by fluorescence detection (left) and SBR-iSCAT (middle) or TD-iSCAT (right) imaging-based cargo localization. The trajectories by iSCAT are superimposed on a snapshot taken at t = 0.0 s. Scale bar: 200 nm. See also Supplementary Figs. 3 and 12.

Supplementary Movie 4. Directional cargo inside a live COS-7 cell. A directionally transported cargo in the crowded cellular environment at the intersection of cytoskeletal highways was visualized in a time-lapse iSCAT movie taken at 50 Hz. Scale bar: 200 nm. See also Fig. 3 (trajectory of the cargo shown in Fig. 3e).

Supplementary Movie 5. Long-term cargo traffic flow revealed by cargo-localization iSCAT. Temporal evolution of cargo traffic flow over 30 minutes was reconstituted by cargo-localization iSCAT microscopy. Each image was acquired at the interval of 60 s and by integrating 9,000 consecutive frames taken at 50 Hz (ΔT = 180 s). The field of view is 20 × 20 μm^2. Scale bar: 1 μm. See also Fig. 2d (white-boxed area in Fig. 2c).

Supplementary Movie 6 and 7. Impact of nocodazole treatment on intracellular traffic in a live COS-7 cell. Temporal evolution of cargo traffic flows reconstituted prior to nocodazole treatment (Movie 6) and after 2 μM nocodazole treatment at t = 20 min (Movie 7). Each image was acquired at the interval of 60 s and by integrating 9,000 consecutive frames taken at 50 Hz (ΔT = 180 s). Scale bar: 2 μm. See also Supplementary Fig.11d.

Supplementary Movie 8. Directionally transported dimeric cargo featuring intermittent pausing (1). A directionally transported dimeric cargo was visualized in time-lapse SBR- and TD-iSCAT movies. The dimeric cargo was intermittently paused by direct contact with a cytoplasmic structure during the time interval ‘a’ in Fig. 4b. Scale bar: 1 μm. See also Fig. 4.

Supplementary Movie 9. Directionally transported dimeric cargo featuring turn-around. A directionally transported dimeric cargo was visualized in time-lapse SBR- and TD-iSCAT movies. The dimeric cargo changed direction due to a head-on collision with another cargo moving in the opposite direction during the time interval 'b' in Fig. 4b. Scale bar: 1 μm. See also Fig. 4.

Supplementary Movie 10. Directionally transported dimeric cargo featuring intermittent pausing (2). A directionally transported dimeric cargo was visualized in time-lapse SBR- and TD-iSCAT movies. The dimeric cargo was intermittently paused again by direct contact with the same cytoplasmic structure during the time interval 'c' in Fig. 4b. Scale bar: 1 μm. See also Fig. 4.

Supplementary Movie 11. Dissociation of a dimeric cargo. The separation of the dimeric cargo was visualized by SBR-iSCAT (left), TD-iSCAT (middle), and fluorescence (right) images taken at 10 Hz. Only the front cargo contains a f-PS bead. See also Supplementary Fig. 15. Scale bar: 1 μm.

Supplementary Movie 12. Directional transport of a trimeric cargo. A directionally transported cargo trimer was visualized in time-lapse TD-iSCAT and fluorescent microscopy movies. The movies were acquired at 10 Hz. Scale bar: 1 μm. See also Supplementary Fig. 16.

Supplementary Movie 13. Directionally transported dimeric cargo featuring various dynamic events. A directionally transported dimeric cargo was visualized in time-lapse movies of 3 different modalities (left: SBR-iSCAT, middle: TD-iSCAT, and right: fluorescent microscopy). The movies were acquired at 10 Hz. Scale bar: 1 μm. See also Supplementary Fig. 17a.

Supplementary Movie 14. U-turn of a dimeric cargo. A U-turn of a dimeric cargo was visualized in time-lapse SBR- (left) and TD-iSCAT (right) images taken at 50 Hz. Scale bar: 200 nm. See also Supplementary Fig. 18.
