# Supplementary material for: Long-term cargo tracking reveals intricate trafficking through active cytoskeletal networks in the crowded cellular environment
Source: Nat Commun. 2023 Nov 14;14:7160. doi: 10.1038/s41467-023-42347-7 (PMC10645962; doi:10.1038/s41467-023-42347-7)
Supplement: Supplementary file 18 — Reporting Summary [file 41467_2023_42347_MOESM18_ESM.pdf]

nature portfolio

Corresponding author(s): Seok-Cheol Hong
Minhaeng Cho

Last updated by author(s): Aug 19, 2023

Reporting Summary

Nature Portfolio wishes to improve the reproducibility of the work that we publish. This form provides structure for consistency and transparency in reporting. For further information on Nature Portfolio policies, see our Editorial Policies and the Editorial Policy Checklist.

Statistics

For all statistical analyses, confirm that the following items are present in the figure legend, table legend, main text, or Methods section.

n/a	Confirmed	
☐	☒	The exact sample size (*n*) for each experimental group/condition, given as a discrete number and unit of measurement
☐	☒	A statement on whether measurements were taken from distinct samples or whether the same sample was measured repeatedly
☒	☐	The statistical test(s) used AND whether they are one- or two-sided<br>*Only common tests should be described solely by name; describe more complex techniques in the Methods section.*
☒	☐	A description of all covariates tested
☒	☐	A description of any assumptions or corrections, such as tests of normality and adjustment for multiple comparisons
☐	☒	A full description of the statistical parameters including central tendency (e.g. means) or other basic estimates (e.g. regression coefficient) AND variation (e.g. standard deviation) or associated estimates of uncertainty (e.g. confidence intervals)
☒	☐	For null hypothesis testing, the test statistic (e.g. *F*, *t*, *r*) with confidence intervals, effect sizes, degrees of freedom and *P* value noted<br>*Give P values as exact values whenever suitable.*
☒	☐	For Bayesian analysis, information on the choice of priors and Markov chain Monte Carlo settings
☒	☐	For hierarchical and complex designs, identification of the appropriate level for tests and full reporting of outcomes
☒	☐	Estimates of effect sizes (e.g. Cohen's *d*, Pearson's *r*), indicating how they were calculated

Our web collection on statistics for biologists contains articles on many of the points above.

Software and code

Policy information about availability of computer code

Data collection	Live cell images were acquired with our home-built fluorescence-combined iSCAT microscopy
Data analysis	OriginPro 2016 were used for all statistical measurements.<br>ImageJ version software 1.52v soft with MosaicSuite ImageJ plugin was used for identification and tracking the individual dynamic cargos moving in cells.<br>iView software (home-built iSCAT image viewer programmed in Matlab R2019b) was used for image processing of raw iSCAT cell images.<br>Particle_Density_Map software (home-built iSCAT data analysis programmed in Matlab R2019b code) was used for analyzing cargo localization density and temporal evolution of cargo flow.<br>SPT_analysis software (home-built iSCAT data analysis programmed in Matlab R2019b) was used for MSD analysis.

For manuscripts utilizing custom algorithms or software that are central to the research but not yet described in published literature, software must be made available to editors and reviewers. We strongly encourage code deposition in a community repository (e.g. GitHub). See the Nature Portfolio guidelines for submitting code & software for further information.

Data

Policy information about availability of data

All manuscripts must include a data availability statement. This statement should provide the following information, where applicable:

- Accession codes, unique identifiers, or web links for publicly available datasets
- A description of any restrictions on data availability
- For clinical datasets or third party data, please ensure that the statement adheres to our policy

All the data supporting the findings of this study are available within the article and its supplementary information files and from the corresponding author upon reasonable request

Field-specific reporting

Please select the one below that is the best fit for your research. If you are not sure, read the appropriate sections before making your selection.

☒ Life sciences ☐ Behavioural & social sciences ☐ Ecological, evolutionary & environmental sciences

For a reference copy of the document with all sections, see nature.com/documents/nr-reporting-summary-flat.pdf

Life sciences study design

All studies must disclose on these points even when the disclosure is negative.

Sample size	All image data presented in manuscript were obtained from a single sample. From the same area of a single cell, about 10-million data points of dynamic cargos were used for data analysis.
Data exclusions	No data was excluded from the analyses.
Replication	Only single experimental set was used for data analysis.
Randomization	There was no grouping of experiments or samples preformed.
Blinding	N/A

Reporting for specific materials, systems and methods

We require information from authors about some types of materials, experimental systems and methods used in many studies. Here, indicate whether each material, system or method listed is relevant to your study. If you are not sure if a list item applies to your research, read the appropriate section before selecting a response.

Materials & experimental systems

n/a	Involved in the study	
☒	☐	Antibodies
☐	☒	Eukaryotic cell lines
☒	☐	Palaeontology and archaeology
☒	☐	Animals and other organisms
☒	☐	Human research participants
☒	☐	Clinical data
☒	☐	Dual use research of concern

Methods

n/a	Involved in the study	
☒	☐	ChIP-seq
☒	☐	Flow cytometry
☒	☐	MRI-based neuroimaging

Eukaryotic cell lines

Policy information about cell lines

Cell line source(s)	COS-7 cell lines, originally purchased from Korean Collection for Type Cultures (KCTC), were donated from Prof. Shim at Korea University.
Authentication	The cell lines were morphologically confirmed according to the information provided by their supplier.
Mycoplasma contamination	All cell lines were tested negative for mycoplasma contamination. No mycoplasma contamination was found before use.

March 2021

Commonly misidentified lines<br>(See ICLAC register)	No commonly misidentified cell lines was used in the study.
